# Controlled three-dimensional polystyrene micro- and nano-structures fabricated by three-dimensional electrospinning†

**DOI:** 10.1039/c7ra13278f

**Published:** 2018-04-24

**Authors:** M. Vong, E. Speirs, C. Klomkliang, I. Akinwumi, W. Nuansing, N. Radacsi

**Affiliations:** The School of Engineering, Institute for Materials and Processes, The University of Edinburgh Robert Stevenson Road Edinburgh EH9 3FB UK n.radacsi@ed.ac.uk; School of Physics, Institute of Science, Suranaree University of Technology Nakhon Ratchasima 30000 Thailand; SUT CoE on Advanced Functional Materials (SUT-AFM), Suranaree University of Technology Nakhon Ratchasima 30000 Thailand

## Abstract

The combination of electrospinning with extrusion based 3D printing technology opens new pathways for micro- and nanofabrication, which can be applied in a wide range of applications. This simple and inexpensive method has been proven to fabricate 3D fibrous polystyrene structures with controlled morphology and micro- to nano-scale fibers diameter. The controllable movement of the nozzle allows precise positioning of the deposition area of the fibers during electrospinning. A programmed circular nozzle pattern results in the formation of controllable 3D polystyrene designed shapes with fiber diameters down to 550 nm. The assembly of the fibrous structures starts instantaneously, and a 4 cm tall and 6 cm wide sample can be produced within a 10 minutes electrospinning process. The product exhibits high stability at ambient conditions. The shape, size, and thickness of fibrous polystyrene structures can be easily controlled by tuning the process parameters. It is assumed that the build-up of 3D fibrous polystyrene structures strongly depends on charge induction and polarization of the electrospun fibers.

## Abstract 2

The combination of electrospinning and extrusion based 3D printing opens new pathways for micro- and nanofabrication in a wide range of applications. The fast production of a highly stable self-standing polystyrene 3D structure is demonstrated.

## Graphical abstract

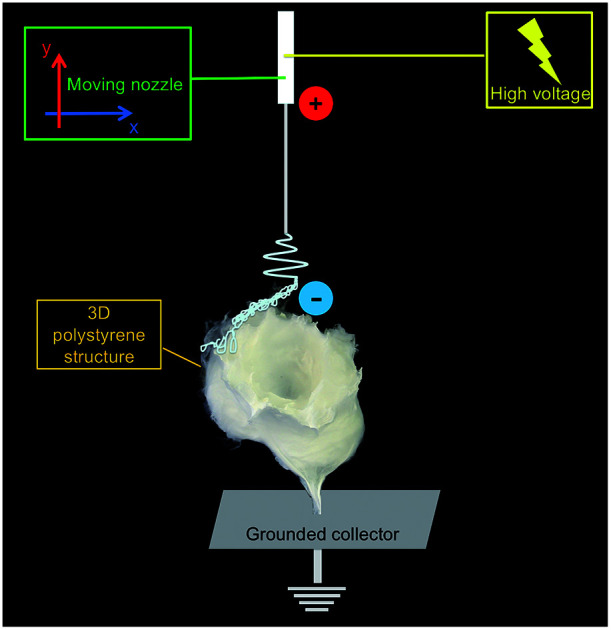

## Introduction

Electrospinning is a simple and efficient process to manufacture micro and nanosized fibers. By applying high voltage to a polymeric solution, a Taylor cone is formed at the tip of a metallic nozzle and nanofibers are ejected from it.^1,2^ Characteristics of the electrospun fibers, such as the nature of the polymer, diameter, surface to volume ratio, porosity, pore-interconnectivity, length, shape and structure, are widely controllable.^3–9^ It is also a versatile technique that allows manufacturing of ceramic and composite fibers.^10–13^ Hence, electrospinning has been researched for different applications in various fields due to its simplicity and promising potentials. Potential applications include areas in biomedicine (*e.g.* wound dressing, bone screw hole and drug delivery), biosensors, cosmetics, protective clothing, catalyst, filtration, adsorption (*e.g.* dye removal and chromatography), batteries and fuel cells.^14–27^

The electrospun fibers are randomly aligned and come in the form of flat two-dimensional (2D) non-woven mats because of the bending instability, inherent to electrospinning.^1,2^ Thus, research has been carried out to circumvent this chaotic 2D deposition. Near-field electrospinning can be used to control the exact positioning of the fibers onto the collector by electrospinning at a short distance between the metallic nozzle and the collector.^28^ With this technique, precise deposition and integration of oriented fibers into functional devices is achievable.^29,30^ A three-dimensional (3D) structure is more desirable than a 2D mat for some applications. A 3D electrospun scaffold is beneficial for bioengineering as it resembles the fibrous structure of the natural extracellular matrix and provides contact guidance for cells.^31,32^ In a particular example, macroscopic 3D electrospun tubes have been shown to be favorable to nervous regeneration without inducing degradation of the nerves due to mechanical stress.^33^ 3D electrospun scaffold has also been constructed to provide good mechanical stress and better cell proliferation and migration in bone tissue engineering.^34^ In other fields of research, it has been used for energy applications, where a composite material was fabricated from 3D carbon nanofibers non-woven,^35^ and filtration purpose, where 3D layering of the nanofibers mat was shown to have an increased filter quality than a single mat.^36^ It is still a challenge to obtain a 3D fibrous macrostructure *via* electrospinning but we can distinguish four main approaches to achieve this.^37^ The main methods are increasing the electrospinning time and stacking multiple layers of fibers,^38^ post-treatment of the 2D non-woven mats,^39^ use of a template-assisted collector^40^ or direct self-assembly of the electrospun polymer.^41–44^ These methods either require several time-consuming steps to obtain the 3D structure or have no control over the final shape of the 3D structure. Electrospinning of designed 3D structures can be achieved by combining traditional electrospinning with the maneuverability of extrusion based 3D printing. By using a nozzle able to move in the *x*–*y* plane, movement and control of the deposition area during the electrospinning process is possible.

In this paper, the fabrication of 3D structures made of polystyrene (PS) fibers using 3D electrospinning, without any auxiliary collecting templates or post-processing steps, is investigated. The study is done by designing a specific nozzle pattern using a computer-aided design (CAD) software, followed by electrospinning of this structure. In this work, the nozzle follows a circular pattern and the desired electrospun structure of reference is a hollow cylinder. The effects of the solution properties, polymer concentration, applied voltage, working distance, flow rate, and nozzle speed on the 3D structures shape and fibers morphology are investigated. It is observed that the improper control of any of these parameters would prevent 3D assembly of the electrospun polymer, and result in a flat 2D deposition. The microscopic properties of the electrospun fibers are investigated *via* Scanning Electron Microscopy (SEM) and the macroscopic shapes of the 3D structures are recorded.

## Experimental

### Materials

The polymer polystyrene (PS) was obtained from Sigma-Aldrich. The weight average molecular weight (*M*_w_) of PS was 280 000. The solvents dimethylformamide (DMF) and tetrahydrofuran (THF) were acquired from Alfa Aesar and Fisher Scientific, respectively. DMF was 99% pure and THF was 99.5% pure. The additives phosphoric acid 85% in water (H_3_PO_4_) and ethanol (99.99%) (EtOH) were received from Acros Organics and Fisher Scientific, respectively.

All products were used without further purification.

### Solution preparation

PS was dissolved in a solvent mixture of 1 : 1 by weight DMF/THF by stirring for 4 hours under ambient conditions. Several concentrations were prepared: 5.0 wt%, 7.5 wt%, 10.0 wt%, 12.5 wt% and 15.0 wt%. An amount of 100 μL of additives were then added (EtOH or H_3_PO_4_) to 50 mL of solution. The different solutions prepared are summarized in Table 1. Each batch started from a fresh solution, and each electrospinning experiment ran for 10 minutes.

**Table 1:** Summary of all electrospun solutions

Composition	PS concentration [wt%]	Additives [100 μL in 50 mL solution]
PS in 1 : 1 DMF/THF	15.0	N/A
	15.0	EtOH
	15.0	H_3_PO_4_
	12.5	H_3_PO_4_
	10.0	H_3_PO_4_
	7.5	H_3_PO_4_
	5.0	H_3_PO_4_

### Electrospinning apparatus

**Fig. 1:**
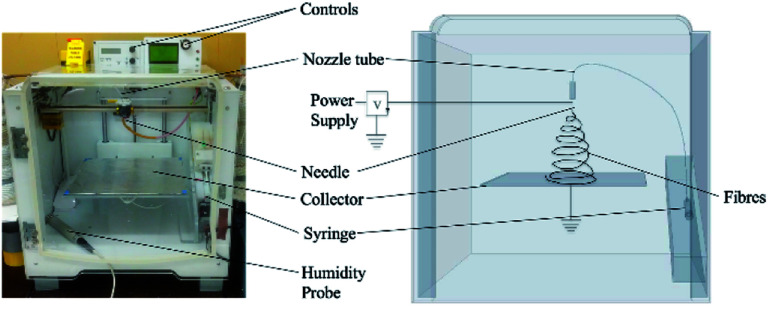
Photo (left) and schematic drawing (right) *Y*–*Z* plane of the 3D electrospinner.

The electrospinning apparatus was combined with extrusion based 3D printing technology to allow for control of various patterns and electrospinning structures. The apparatus itself (see Fig. 1) (NovaSpider, CIC nanoGUNE, Spain) consists of a syringe pump, a nozzle and a high voltage DC power supply. The nozzle is capable of movements in the *x*–*y* axis, with a resolution of 0.02 mm, while the collector (print bed) can be set along the *z*-axis. In this study, the nozzle head was moving along the *x*–*y* axis while the collector plate was fixed along the *z* axis during the experiment, but could be changed at will. Therefore, the electrospinning process here will be referred to as ‘3D electrospinning’.

The collector plate held a sheet of aluminum foil connected to the ground, and possibly to a negative voltage supply. The solution was loaded into a syringe whose needle was connected to the positive power supply and had an inner diameter of 0.603 mm. All experiments were carried out at room temperature, between 20–27 °C, and the relative humidity, measured by a temperature and humidity sensor (HumidiProbe, Pico Technology, United Kingdom) was between 45–55%.

### 2D pattern design: gcode/slicer

**Fig. 2:**
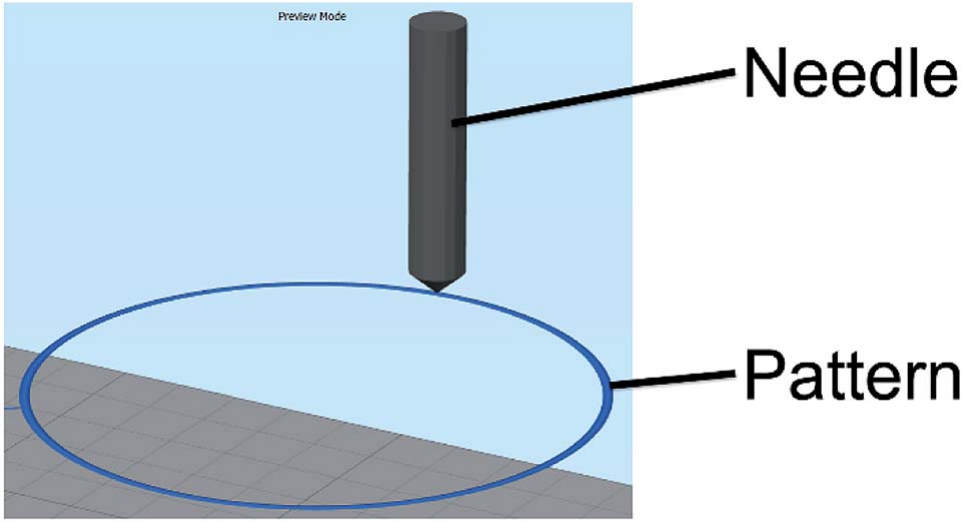
Pattern of the nozzle as seen by the 3D printing slicing software (Simplify3D). A circle of 5.5 cm was designed as the nozzle pattern.

The nozzle pattern was generated by using a Computer Aided Design (CAD) software (Onshape). The designed pattern was a hollow cylinder of 5.5 cm. The model was exported as an STL file. The model was then processed with a 3D printing slicing software (Simplify3D) to get a gcode file readable by the 3D electrospinner. The gcode is controlling the pattern of the nozzle, the moving speed of the nozzle, the working distance and the flow rate of the syringe. The applied voltage to the nozzle was controlled on a separate power supply. The pattern (the movement of the nozzle) was first fixed to be a circular motion of 5.5 cm, as seen in Fig. 2, and the time of experiment to 10 minutes.

### Characterization

#### Digital camera

The macroscopic shape of the 3D structure after electrospinning was captured using a digital camera (EOS 6D, Canon Inc., Japan). The electrospinning process of the 3D structures was captured with the same camera.

#### Scanning electron microscope (SEM)

The shape and size of the electrospun PS fibers were observed with a Scanning Electron Microscope (SEM) (JSM-6010PLUS/LV, JEOL Ltd., Japan) at an accelerating voltage of +20 kV. Prior to observation, the samples were coated with 20 nm of gold using a Sputter coater (Desk III, Denton Vacuum, USA). The diameters of the fibers were measured with an image processing program (Fiji – ImageJ). The mean diameter was typically taken using more than 100 fibers per sample.

## Results and discussion

### Formation mechanism of the 3D structure

The incorporation of additives during the electrospinning of polystyrene fibers is essential for obtaining 3D structures. Under ambient conditions, PS solutions without additives produce flat, 2D structures with electrospinning.^41^ Adding EtOH, as suggested by Sun *et al.*,^42^ still resulted in flat structures.

As previous studies have indicated, the mechanism behind the self-assembly of electrospun fibers is based on the rapid solidification of the fibers, which allows the structure to be self-standing, and the polarization and electrostatic induction of the deposited fibers.^42,45^ The top of the deposited mat gets negatively charged because of the strong electric field. This negative top then becomes a preferential deposition site and attracts the positively charged jet coming out of the nozzle. As the fibers at the top of the layer have the same charge, they would naturally repel each other during the build-up and fabricate a non-compact structure. Using additives into the solution can increase the polarizability of the electrospun fibers, leading to repulsive forces between fibers. As H_3_PO_4_ is more conductive than EtOH,^46,47^ this would explain why the solution doped with H_3_PO_4_ gives 3D structures, while the solutions doped with EtOH or without any doping agent result in 2D (flat) structures. Therefore, H_3_PO_4_ was used in this study as a doping agent. The static induction and polarization effect can be proven by using a charged rod to attract or repel the electrospun fibers. This test was done under two different conditions: after electrospinning, when the structure was already built up with the electric field turned off, and during the electrospinning process, when the high voltage was still applied on the moving nozzle. The test after electrospinning was carried out on a sample that was electrospun for 3 minutes. It was observed that both a positively and negatively charged rod would attract the top of the deposited fiber mat. This is in contrast with the work of Sun *et al.*,^42^ who stated that the fibers would be attracted to the positive rod but repelled by the negative rod. This difference in behavior might be because the rods used in our study had a high voltage (±5 kV), powerful enough to induce a charge on the 3D structure. It was also observed that the electrospun structures were barely attracted or repelled by a charged rod twelve hours after the experiment. This could be another explanation for the difference in results with previous works. This result shows the ease of inducing a charge on the fibers, during or right after electrospinning. In the second test, the charged rod was placed about 1.5 cm above the collector, under the way of the circular pattern of the nozzle. Despite the additional charged rod, it was observed that the 3D structures would still be built up along the circular pattern of the nozzle. The electrospun fibers reacted to the charged rod only when close to its vicinity. They were attracted to the positively charged rod, being compacted to the rod and slowly pulling it toward the collector where a short circuit would then occur (see video in ESI†). The fibers could still be deposited onto the negatively charged rod however, they would not be compacted on the rod, but rather self-standing as they were repelled because of their similar charge with the negative rod. This test confirms that during electrospinning, the top of the 3D structure, including the fibers that were just electrospun, are immediately induced with a negative charge because of the high positive voltage at the nozzle. This explains why the charge induction effect is not limited to the first layer of electrospun fibers and why the 3D structures can be built up relatively high.

**Fig. 3:**
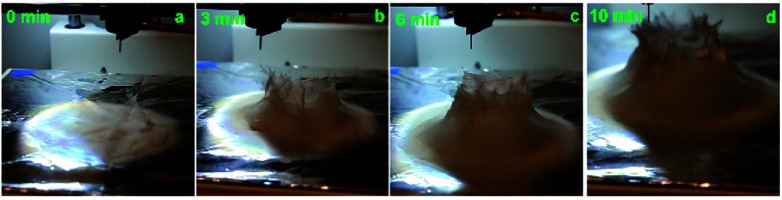
Growth process of the electrospun PS fibers 3D structure. The electrospinning was performed at a voltage of +12 kV, a working distance of 5 cm, a flow rate of 5.0 mL h^−1^ and at a nozzle moving speed of 12.0 mm s^−1^ for 10 minutes. The 3D build-up starts after an initial flat layer of fibers have been deposited onto the collector as seen in (a). The build-up follows the circular pattern of the nozzle, forming a hollow cylinder. (b)–(d) The 3D structure is already observable after 3 minutes and the cylinder shape is followed during the whole 10 minutes of electrospinning.

**Fig. 4:**
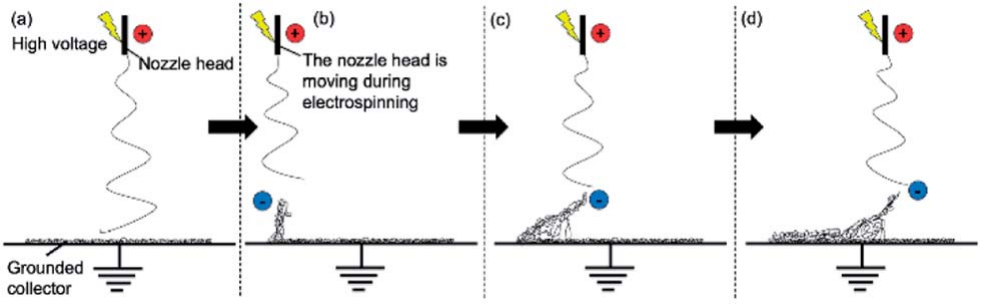
Simplified formation mechanism of the 3D structure. (a) An initial layer of fibers covers the grounded collector. (b) The negatively induced fibers repel each other and are attracted by the positively charged nozzle; thus the build-up of the structure. (c) The negative fibers branches are dragged along the nozzle-pattern. Some fibers are growing downwards the previous layer and stick to it. (d) The fibers far away from the nozzle are no longer attracted to it; thus the fibers settle and consolidate the structure.

The growth process of the electrospun 3D structure was recorded using a digital camera. Pictures at different time interval are shown in Fig. 3, more time intervals can be seen in the ESI S1† and its associated video. The 3D build-up of electrospun fibers starts only after an initial layer of flat fibers has been deposited onto the collector, which is in line with another study reported in literature.^43^ This initial layer is necessary to prevent the following electrospun fibers to touch the grounded collector and neutralize the pulling force of the collector towards the charged fibers. It is also from this first layer that the polarization and charge induction effect can start. In our case, the initiation of the 3D build-up can be seen a few seconds after the start of this experiment. Then, similar to the self-assembly of PS fibers electrospun by Li and Long, the 3D structure grew up in small branches.^41^ The difference in our experiment was that the nozzle was moving in a circular pattern. As such, the branches, attracted by the positively charged nozzle, were dragged along the circular pattern of the nozzle and some fibers were growing downwards toward the previous layers of the 3D structure and sticking to it. The weight of the fibers also played a role in compacting the 3D structure. This is how the structure is built up as a bulk, instead of a single thread of fibers wrapped over itself. A fast growth of the wall thickness of the cylinder is observed within the first few layers of fibers. This is because the already deposited self-standing layers of polymer increase the amount of fibers that is able to be built up. These self-standing layers, being negatively induced, also promote repulsion between fibers. As a result, the effective surface covered by the fibers increases once they settle onto the 3D structure. The increase in thickness of the cylinder is limited by the weak whipping instability due to the fast solidification of the polymer fibers. As seen in the ESI S2,† the wall thickness increased from 0.8 cm, in the first layer of self-standing fibers, to around 2.3 cm in the 5^th^ layer. The final thickness after 10 minutes of electrospinning, about 40 layers, was 3.2 cm. Furthermore, the electric field at the collector is the highest at the point directly under the nozzle. This, along with the fact that the deposited fibers are negatively polarized, explains why the 3D structure is able to be built-up along the shape of the nozzle pattern. The whole formation mechanism is illustrated in Fig. 4.

**Fig. 5:**
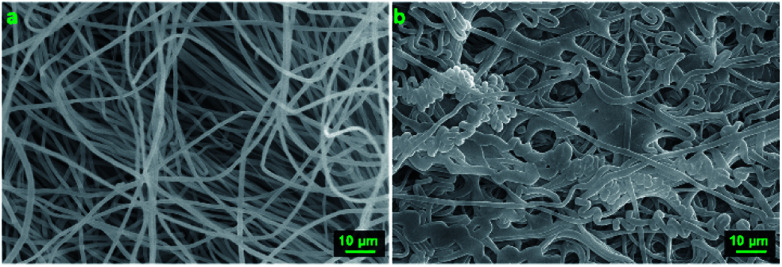
SEM pictures of the sample shown in Fig. 3. (a) Fibers taken from the inside lower part of the 3D structure. (b) Fibers taken from the top of the 3D structure, much closer to the nozzle tip.

In a 10 minutes experiment at a working distance of 5 cm, the 3D structures could be as high as 4 cm. A direct implication of this is, as the 3D structure gets build-up, the top part gets closer to the charged nozzle and is subjected to a higher electric field. This further enhance the charge induction and polarization effect and favor the deposition of fibers on the grown structure rather than the flat collector. However, this also mean the fibers getting at the top of the structure would have less travel length and time to dry. This can be directly observed by comparing the shape and morphology of the fibers at the inner bottom and at the top of the 3D structure as seen in Fig. 5. The lower part of the structure is spongy, soft and made of single, randomly oriented fibers, while the top of the structure is brittle and made of fused fibers. Another consequence of the weaker whipping instability is the wall thickness of the cylinder at the top part, which is lower than 0.1 cm. The resulting 3D structures are self-standing even after 6 months of storage at ambient conditions. The experiments were done at ambient conditions, at a relative humidity of 45–55%.

The following process parameters were identified to be key for the 3D electrospinning: solution concentration, applied voltage, working distance, flow rate and nozzle moving speed. It is not possible to obtain a structure shaped exactly like the nozzle pattern because of the whipping instability, inherent to electrospinning. The parametric study aimed to optimize the shape of the final 3D structure, so that it was as close as possible to the nozzle pattern, a 5.5 cm hollow cylinder. The criteria for a good sample were based on the overall shape itself (distorted cylinder or not), the height of the structure, whether or not the structure was hollow, the speed of building up and to some extent, the morphology of the fibers.

### Solution concentration (*c*)

Five different solution concentrations have been electrospun at a working distance of 5 cm, a voltage of +20 kV, a flow rate of 5.0 mL h^−1^ and a nozzle speed of 12.0 mm s^−1^ at ambient conditions. H_3_PO_4_ was added in a 0.2 v/v% volume ratio. The PS concentrations were 5.0 wt%, 7.5 wt%, 10.0 wt%, 12.5 wt% and 15.0 wt%. In that range of concentration, the electrospinning jet was stable and no solution dripping was observed.

**Fig. 6:**
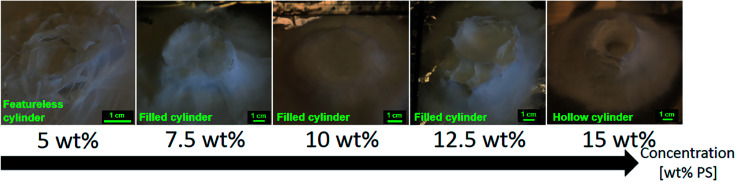
Evolution of the 3D structures shape as the weight concentration of PS is increased from 5.0 wt% to 15.0 wt%. The solutions were electrospun at a voltage of +20 kV, a working distance of 5 cm, a flow rate of 5.0 mL h^−1^ and at a nozzle speed of 12.0 mm s^−1^ for 10 minutes. No 3D build-up were observed at low polymer concentration. The shape of the 3D structure is influenced by the polymer concentration.

The 15.0 wt% PS solution is the only one that would give a cylinder shape with a hollow inside. 7.5 wt%, 10.0 wt% and 12.5 wt% would give a full cylinder shape rather than a hollow cylinder. Mit-Uppatham *et al.* explained a higher solution concentration, and thus viscosity, would give the electrospun jet greater resistance toward thinning of its diameter.^48^ This would result in a longer straight jet trajectory before the bending instability and would in turn result in a smaller deposition area. This explains why only the higher concentrated PS solution could have a hollow cylinder shape. The other lower concentrations had a deposition area big enough to partly cover the inside of the cylinder. Another consequence of the wider deposition area was the final height of the 3D structure after 10 minutes of electrospinning. The 3D structures electrospun with 7.5 wt% and 10.0 wt% PS were smaller in height than the ones prepared with 12.5 wt% and 15.0 wt% PS. Another explanation would be the lower amount of polymer provided to the 3D structure, which would effectively result in a smaller 3D structure after the same time of experiment. The top of the smaller 3D structures was also not brittle, as depicted in Fig. 6, as the travel length was still sufficient for the fibers to dry properly. At the lowest concentration of 5.0 wt%, the electrospun 3D structures had no features. It is necessary to provide a significant amount of fibers at once to get a proper 3D build-up of the PS solution. If not, the charge induction and polarization effect would not be strong enough and this would result in a flat deposition of the electrospun fibers, as there would be no repulsion between fibers to get the 3D build-up. The resulting deposition area was bigger than for the higher concentrations. As PS is a poor conductor, some charges were still retained by the deposited fibers and diverted the electrospinning jet to regions of lower resistance.^49^ The overall shapes of the 3D structures electrospun with different PS concentration solutions is summarized in Fig. 6.

**Fig. 7:**
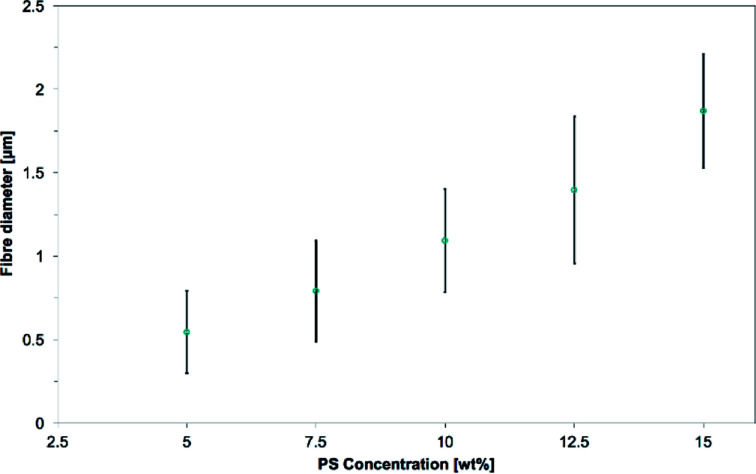
Evolution of the mean fiber diameters as the PS concentration is increased from 5.0 to 15.0 wt% in 1 : 1 DMF/THF. The electrospinning parameters were a voltage of +20 kV, a working distance of 5 cm, a flow rate of 5.0 mL h^−1^ and a nozzle moving speed of 12.0 mm s^−1^ for 10 minutes. Mean fiber diameter increases with the polymer concentration.

The mean fiber diameter increases with polymer concentration as seen in Fig. 7, the lesser concentrated PS solution giving smaller fibers. The mean diameters as measured on SEM are 0.55 ± 0.25 μm, 0.79 ± 0.30 μm, 1.09 ± 0.31 μm, 1.40 ± 0.44 μm and 1.87 ± 0.34 μm for the 5.0 wt%, 7.5 wt%, 10.0 wt%, 12.5 wt% and 15.0 wt% PS solution respectively. This trend has been observed several times by other researchers.^50–52^ The electrospun jet of the lesser concentrated solutions could stretch and thin more because of the lower viscosity, resulting in smaller fibers. Even though the concentrations of 5.0 wt% and 7.5 wt% had fibers diameters in the sub-micron scale, these electrospun solutions gave mixtures of fibers and beads. This trend has been already observed,^53,54^ and as Lee *et al.* suggested, it might be due to the lower solution viscosity that leads to a less stable jet formation.^55^ SEM pictures of the samples are available in the ESI S3.†

The 15.0 wt% PS solution was selected, as it was the only solution yielding a hollow cylinder as designed in the nozzle pattern.

### Applied voltage (*V*)

The effect of the applied voltage on the 3D structure was tested in a range of 6 kV to 20 kV, with increments of 1 kV. All the other parameters were fixed. The working distance was 5 cm, the flowrate was 5.0 mL h^−1^, the nozzle speed was 12.0 mm s^−1^, and 15.0% PS in 1 : 1 DMF/THF doped with H_3_PO_4_ was electrospun at ambient conditions. Examples of electric field simulations are shown for applied voltages of 7, 15 and 20 kV in the ESI S4.†

The electrospinning jet was not stable at +6 kV, resulting in lots of dripping of the solution. The 3D structures and stable electrospinning were obtained when the applied voltage was higher than +7 kV.

A few differences were observed with the final shape and height of the resulting cylinders after electrospinning. Only the sample made at +10 kV seemed to show a shape closer to the designed 5.5 cm diameter hollow cylinder, as its outer wall was thinner and vertical. However, the time needed to go from a flat electrospinning to a clear 3D build-up decreased as the applied voltage increased. Typically, the 3D build-up would happen in less than 20 seconds when the applied voltage was higher than +15 kV and a flat electrospinning time of 4 minutes 30 seconds was necessary before the 3D structuring was observed at the voltage of +8 kV. As explained earlier, the 3D build-up is due to the polarization and static induction of the deposited mat. If a lower voltage is applied, more time would be necessary for the top of the deposited mat to get properly polarized, acquire enough negative charges to attract the positively charged jet, and thus build-up the 3D structure.

**Fig. 8:**
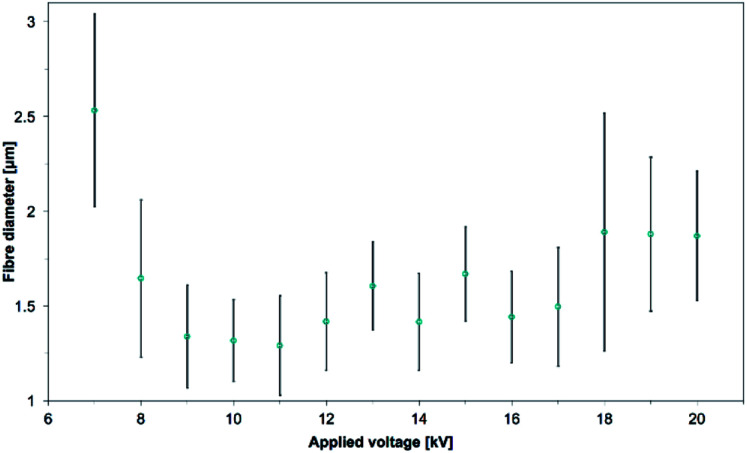
Evolution of the mean fiber diameters as the voltage is increased from +7 kV to 20 kV. 15.0 wt% PS in 1 : 1 DMF/THF was electrospun at a working distance of 5 cm, a flow rate of 5.0 mL h^−1^, at a nozzle moving speed of 12.0 mm s^−1^ for 10 minutes. There was a critical voltage at which point the mean fiber diameter would increase again, up to a stable range after 18 kV.

The electrospun polymer, under SEM observation, showed a similar fibers shape but different fibers diameters. The measured mean diameters were 2.53 ± 0.51, 1.65 ± 0.42, 1.34 ± 0.27, 1.32 ± 0.22, 1.29 ± 0.26, 1.42 ± 0.26, 1.61 ± 0.23, 1.42 ± 0.26, 1.67 ± 0.25, 1.44 ± 0.24, 1.50 ± 0.31, 1.89 ± 0.63, 1.88 ± 0.41, 1.88 ± 0.34 μm for the applied voltage from 7 to 20 kV, respectively (see Fig. 8). The effects of applied voltage on the fiber diameters is one of the most controversial and contradicted in electrospinning experiments. Electrospun fibers have been observed to be thicker with both increasing voltage^56–58^ and decreasing voltage^59,60^ or having a critical voltage value where the trend would be reversed.^61^ The applied voltage influences the electric field, which can have multiple effects on the electrospun jet. In this study, at the working distance of 5 cm, there was no linear trend between the mean fiber diameter and the applied voltage. Instead, the fiber diameter was decreasing until the voltage of +11 kV where it would then increase up to a stable range at voltages higher than +18 kV. This would mean that up to +11 kV, increasing the voltage results in a stronger electric field which would lengthen the elongation of the jet and decrease the fiber diameters, by allowing more stretching and splitting of the fibers.^62^ Further increasing the voltage up to +18 kV could result on a decrease of the size of the initial Taylor cone or a higher jet velocity. These two reasons together can counteract the effect of the lengthened jet and lead to bigger fibers. At voltages between 18 kV and 20 kV, the above mentioned effects might cancel out each other.

**Fig. 9:**
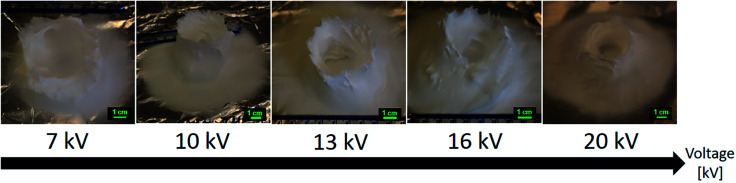
Evolution of the 3D structures shape as the voltage is increased from +7 kV to +20 kV. 15.0 wt% PS in 1 : 1 DMF/THF was electrospun at a working distance of 5 cm, a flow rate of 5.0 mL h^−1^, at a nozzle moving speed of 12.0 mm s^−1^ for 10 minutes. The sample processed at +10 kV had the closest representation to the designed cylinder, having thin walls and the least amount of fibers coverage inside the cylinder. Other than that, the applied voltage had little influence on the cylinder shape.

As minimal differences were observed with the final shape of the electrospun cylinder (see Fig. 9), the high voltage of +20 kV was chosen to minimize the time necessary before the start of the 3D build-up. This high voltage was also selected to make sure a stable electrospinning process could be obtained for further studies that would have required a higher working distance or a higher flow rate. Pictures and fibers morphology of the samples electrospun at the other applied voltage are in the ESI (Fig. S5 to S7†).

### Working distance (WD)

Investigations on the influences of working distance on the 3D build-up were done at a flow rate of 5.0 mL h^−1^ and a nozzle speed of 12.0 mm s^−1^. The voltage was fixed at +20 kV for most distances except for the low working distance of 1 cm. A voltage of +10 kV was used for the working distance of 1 cm, this was necessary to avoid short circuit between the charged nozzle and the collector plate.

**Fig. 10:**
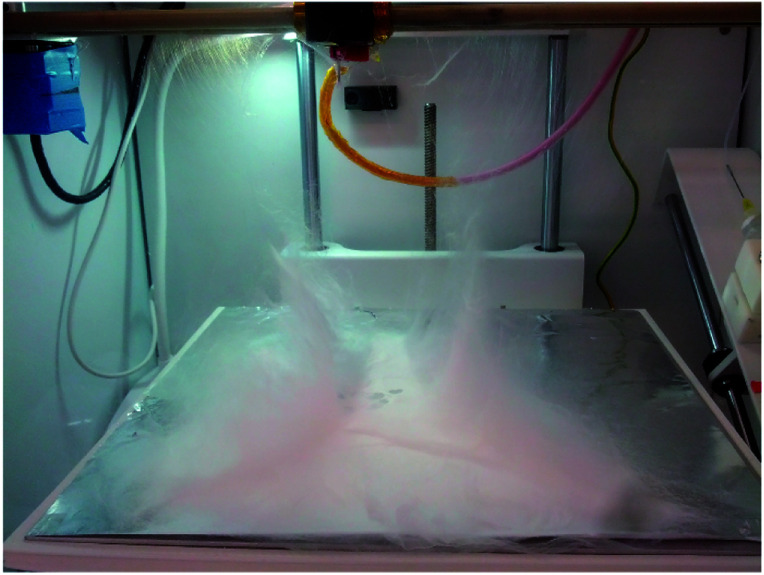
At a working distance of 10 cm, the electrospun fibers were attracted to the rubber belt of the moving nozzle. 15.0 wt% PS in 1 : 1 DMF/THF was electrospun at 20 kV, a flow rate of 5.0 mL h^−1^, at a nozzle moving speed of 12.0 mm s^−1^, at ambient conditions.

Working distances above 10 cm would not result in a stable 3D build-up. At WD 10 cm, a fibers network between the moving nozzle belt and the collector plate is observed (see Fig. 10). At WD 15 cm, the fibers were only deposited on the nozzle collecting belt, and no structures were observed on the collector plate. At high working distance, the nozzle collecting belt can act as a preferential collector because of its closer proximity to the nozzle tip. Then, the electrospun fibers deposited onto the collecting belt can get negatively polarized and act as a preferential deposition sites. Leaving the electrospinning for too long in that condition would result in a short circuit inside the device.

Working distances below 2 cm were not yielding 3D structures. Below WD 2 cm, sparks are likely to occur due to the high electric field. Furthermore, the drying of the electrospun fibers is not complete for working distances under 2 cm. This results in a deposition of wet fibers onto the collector that would then merge and form a solid brittle deposition. It is worth noting that the deposition area of the fibers was smaller at lower working distances. At low working distances, the electrospinning jet is at the early stage of the whipping instability and the jet cone spread is still small.^63,64^

**Fig. 11:**
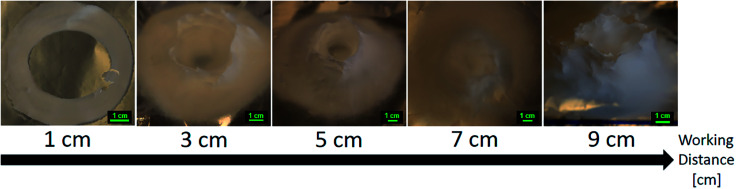
Evolution of the 3D structures shape as the working distance is increased from 1 cm to 9 cm. 15.0 wt% PS in 1 : 1 DMF/THF was electrospun at a voltage of +20 kV, a flow rate of 5.0 mL h^−1^, at a nozzle moving speed of 12.0 mm s^−1^ for 10 minutes. At low working distances and ambient conditions, flight time of the electrospun jet was insufficient and fibers were fused together. On the contrary, increasing the working distance too much is detrimental to the overall shape of the electrospun cylinder.

For intermediate working distance, between 3 and 7 cm, a 3D build-up, with proper shaping of the cylinder, was observed. This range of working distance can be seen as a transition between the low and high working distance. In effect, cylinders electrospun at WD 3 cm and 4 cm were small and most of the top part were constituted of solid brittle fibers that did not have enough travel time to dry. The shape of the build-up was still a hollow cylinder, which again is because of the lower whipping instability at low working distance. On the other end, increasing the working distance too much would decrease the quality of the cylinder shape obtained, resulting in a filling of the hollow inside of the cylinder or a more distorted cylinder. Cha *et al.* observed that the deposition area of their electrospun polymer fibers would increase as the working distance is increased.^65^ This is because of the bending and whipping instability, characteristic of the electrospun jet, which gets wider and wider as more travel time and length is given to the jet. The structure electrospun at a working distance of 6 cm did not have a hollow inside. The shape of the samples electrospun at WD 8 and 9 cm was closer to an elliptic cylinder than a circular one. It is possible to define a major and a minor axis for these elliptic samples by measuring the shortest and longest inner diameters. For example, the sample electrospun at WD 9 cm (see Fig. 11) had a major axis of 82 mm and a minor axis of 64 mm. As seen previously, the nozzle collecting belt can act as a deposition site when the working distance is too high. At these working distances, the presence of the nozzle collecting belt influenced the electric field and altered the travel path of the electrospun jet, resulting in a distorted, elliptic shape.

**Fig. 12:**
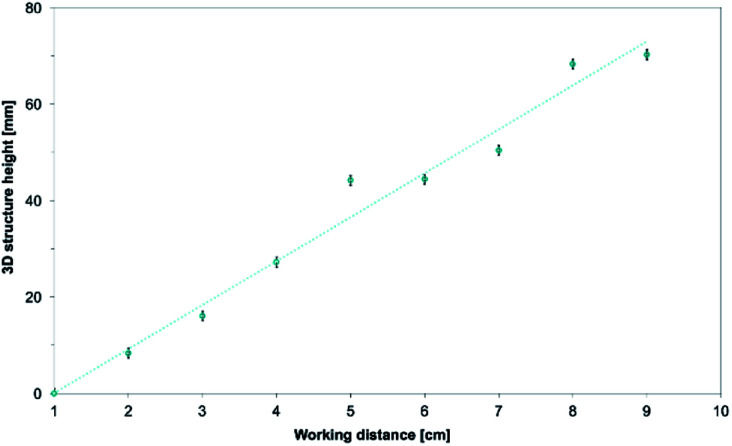
Evolution of the 3D structures height as the working distance is increased from 1 cm to 9 cm. 15.0 wt% PS in 1 : 1 DMF/THF was electrospun at a voltage of +20 kV, a flow rate of 5.0 mL h^−1^, at a nozzle moving speed of 12.0 mm s^−1^ for 10 minutes. The working distance had a linear correlation with the height of the electrospun 3D structures.

The final height of the 3D structure is directly correlated to the working distance as seen Fig. 12. As explained before, the top of the structure is made of fused and brittle fibers because of the lessened travel and drying time. Because these fibers are fused together, the negatively charged fibers are unable to repel each other. This effect stalls the build-up process of the 3D structure. As an example, the height of the 3D structures goes from ∼16 mm to ∼70 mm when the working distance is increased from 3 to 9 cm.

**Fig. 13:**
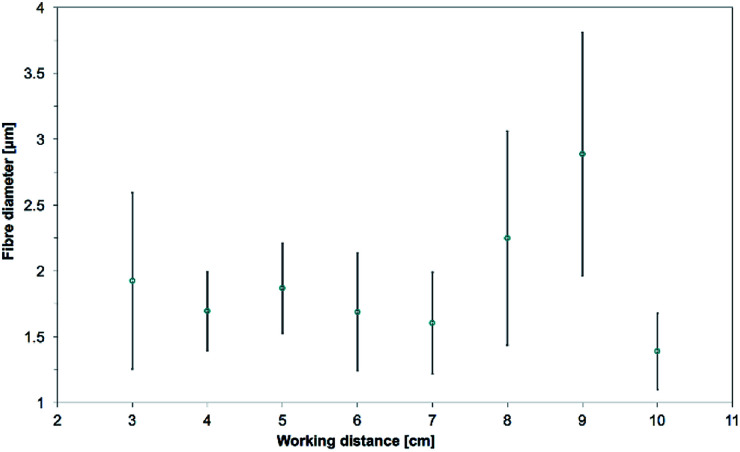
Evolution of the mean fiber diameters as the working distance is increased from 3 cm to 10 cm. 15.0 wt% PS in 1 : 1 DMF/THF was electrospun at a voltage of +20 kV, a flow rate of 5.0 mL h^−1^, at a nozzle moving speed of 12.0 mm s^−1^ for 10 minutes. The working distance had no linear correlation with the mean fiber diameter.

The measured mean fiber diameters were 1.92 ± 0.67 μm, 1.69 ± 0.30 μm, 1.87 ± 0.34 μm, 1.69 ± 0.45 μm, 1.60 ± 0.39 μm, 2.25 ± 0.81 μm, 2.89 ± 0.92 μm and 1.39 ± 0.29 μm for working distances of 3 cm, 4 cm, 5 cm, 6 cm, 7 cm, 8 cm, 9 cm and 10 cm respectively (see Fig. 13). The size of the electrospun samples at 1 and 2 cm were not measured as they were mostly made of fused fibers. As a general behavior in electrospinning, it has been observed that increasing the working distance would result in a decrease of the fibers diameter. The main reason for this was the increased travel length of the jet which would allow better drying and more stretching and thinning of the fibers.^59^ However, beaded fibers and non-smooth fibers have been observed when the working distance was too high.^5^

At 8 and 9 cm, this trend is not observed. This is because even though the applied voltage was the same, the electric field was weakened and this led to a reduction of the stretching of the jet. Bosworth *et al.* observed this behavior for high working distance and obtained higher fibers diameter.^66^ Tong and Wang also explained that a higher working distance would not necessarily lead to a longer travelling distance.^67^ This is due to the three-dimensional spiraling trajectory of the jet, where the travel length of the jet is not dependent only on the height and can be significantly increased within the normal plane. At WD 10 cm however, the fibers diameters are at the lowest of the working distance study. In the case of this study, the travel path of the fibers that reached the collector may have been much longer with the working distance of 10 cm. This can be hinted by the size of the deposition area, which was much larger than in other experiments, and covered about 3/4 of the collector plate (about 20 cm × 20 cm). The increased travel length would thus result in a decrease of the fiber diameters.

A working distance of 5 cm was selected as the optimal distance as it gave the most accurate cylinder and the obtained fibers diameter was among the lowest. Pictures and fibers morphology of the samples electrospun at the other working distances are in the ESI (Fig. S8 to S10†).

### Solution flow rate (*f*)

**Fig. 14:**
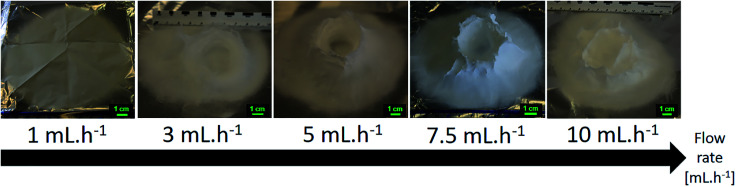
Evolution of the 3D structures shape as the flow rate is increased from 1.0 mL h^−1^ to 10.0 mL h^−1^. 15.0 wt% PS in 1 : 1 DMF/THF was electrospun at a voltage of +20 kV, a working distance of 5 cm, at a nozzle moving speed of 12.0 mm s^−1^ for 10 minutes. A minimum flow rate is necessary to allow 3D build-up to happen. Increasing the flow rate too much hinders the controlled build-up of the 3D structure.

Flow rates of 1.0, 2.0, 3.0, 4.0, 5.0, 7.5, 10.0 and 20.0 mL h^−1^ were tested at a working distance of 5 cm, a voltage of 20 kV and a nozzle speed of 12 mm s^−1^. Using low flow rates has similar effect on the 3D build-up than using low polymer concentration solutions. In both case, a low amount of polymer fibers is deposited onto the collector. As seen in Fig. 14, the lowest flow rates of 1.0 mL h^−1^ would not yield any 3D structure. The flat electrospun mat done at 2.0 mL h^−1^ can be seen in the ESI (Fig. S11†). This might be because of the lesser amount of fibers getting negatively charged by static induction and polarization and this lower amount of negatively charged fibers cannot act as a preferential collector site for the incoming electrospun fibers. Instead, a wider flat deposition area was observed which is characteristic of charge retention by the fibers mat which repels the electrospun jet.^49^ For flow rate of 3.0 and 4.0 mL h^−1^, it was observed that the 3D structures got an overall lower height after the same time of experiment. Similar to the effect of low polymer concentration, less polymer fibers would result in less repulsion between fibers and a lower quantity of fibers to build up the structure.

At flow rates higher than 7.5 mL h^−1^, the structure of the cylinder is also questionable as the inside of the cylinder is partly covered. Deitzel *et al.* noticed at high flow rate, for low molecular weight polymer, the jet radius would shrink slowly.^68^ As such, because the initial jet is wide enough to cover the inside of the ring, if the jet radius shrinks at a slower pace, then the inside of the ring would be covered with more fibers. This would then result in a non-hollow cylinder. The drying of the fibers is another problem at a high flow rate even though no dripping was observed during electrospinning. A significant part of the resulting cylinders electrospun at 10.0 mL h^−1^ and 20.0 mL h^−1^ are made of a hard crest of non-dried polystyrene, similar to when the 3D structure gets too close to the nozzle tip (see Fig. 5). This hard crest would cover the whole surface of the 3D structure, even though the total height of the structure is smaller than 3 cm.

**Fig. 15:**
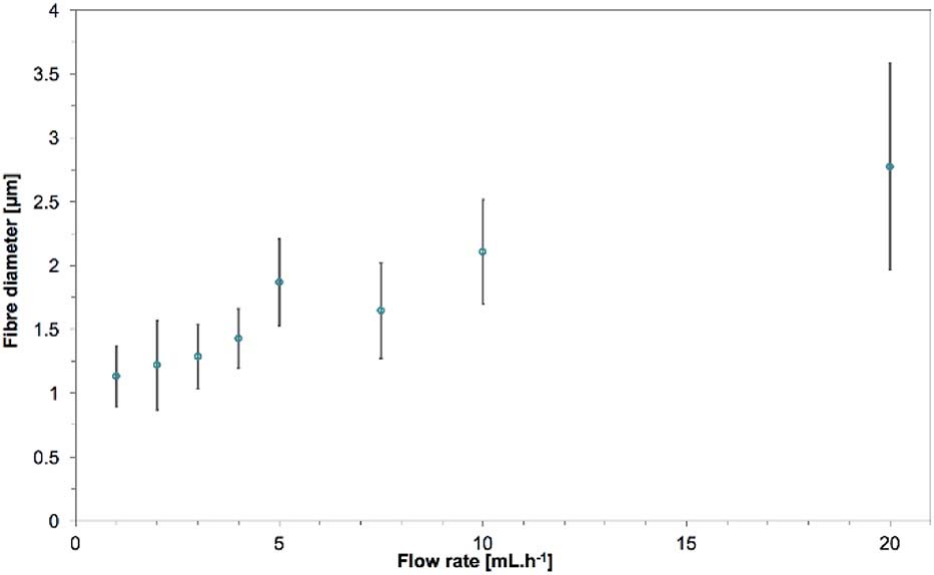
Evolution of the mean fiber diameters as the flow rate is increased from 1.0 to 20.0 mL h^−1^. 15.0 wt% PS in 1 : 1 DMF/THF was electrospun at a voltage of +20 kV, a working distance of 5 cm, at a nozzle moving speed of 12.0 mm s^−1^ for 10 minutes. Mean fiber diameters increase with the flow rate, the amount of polymer supplied.

The mean fiber diameters were 1.13 ± 0.24, 1.22 ± 0.35, 1.29 ± 0.25, 1.43 ± 0.23, 1.87 ± 0.34, 1.65 ± 0.37, 2.11 ± 0.41, 2.78 ± 0.81 μm for flow rates of 1, 2, 3, 4, 5, 7.5, 10 and 20 mL h^−1^ respectively (see Fig. 15). As Zargham *et al.* explained, higher flowrates led to a greater volume of solution being ejected from the needle tip.^69^ This higher volume of solution would also need a longer time to dry and the jet would need more stretching to achieve a lower diameter. Considering the applied voltage and working distance were the same, the travel time was also the same in the flow rate study. This would explain why increasing flow rates lead to an increase in the mean fiber diameter. They further stated that increasing the flow rate at a constant voltage would be detrimental as the amount of charged ions would not be enough for sufficient stretching of the solution.

Even though higher flow rates would give higher production rates, the flow rate of 5.0 mL h^−1^ was chosen from this point to allow better control of the 3D structure and the fibers morphology. Pictures and fibers morphology of the samples electrospun at the other flow rates are in the ESI (Fig. S11 and S12†).

### Nozzle moving speed (*N*_s_)

Investigations on the effects of the moving speed of the nozzle on the 3D structure were done at a fixed voltage of +20 kV, a working distance of 5 cm and a flow rate of 5.0 mL h^−1^. The default moving speed of the nozzle was set to 12.0 mm s^−1^ (default value of the software).

**Fig. 16:**
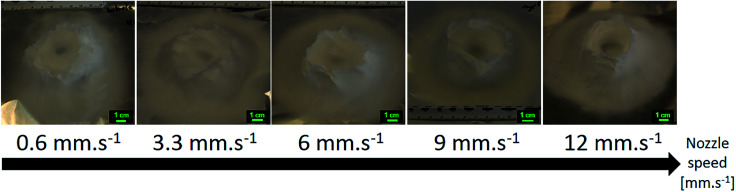
Evolution of the 3D structures shape as the nozzle speed is increased from 0.6 mm s^−1^ to 12.0 mm s^−1^. 15.0 wt% PS in 1 : 1 DMF/THF was electrospun at a voltage of +20 kV, a working distance of 5 cm and a flow rate of 5.0 mL h^−1^ for 10 minutes. Low nozzle speed was detrimental to both the 3D structure.

Several slower speeds were tested (0.6, 3.3, 6.0 and 9.0 mm s^−1^). As the speed was lowered, the shape of the final 3D structure got further away from the designed cylinder. Evidences of discrepancies appeared as more filling of the inside of the cylinder and not vertical building of the outer walls of the cylinder as seen in Fig. 16. It is worth noting that this experiment was also tried with a static single-nozzle electrospinning device under the same experimental conditions: a flow rate of 5.0 mL h^−1^, a voltage of +20 kV, a working distance of 5 cm and a nozzle moving speed of 0 mm s^−1^. The fibers were observed to build-up as a single branch until they touched the nozzle at which point the electrospinning would stop and dripping of the solution would occur. This building up happens in a few seconds.

A doubled moving speed of 24.0 mm s^−1^ resulted in an overall smaller 3D structure of about 2–3 cm high, instead of 3–4 cm. No matter the nozzle moving speed, the negatively induced fibers are attracted by the positively charged nozzle and are dragged along the pathway of the nozzle. At high moving speed, the growing branches are forced onto a smaller slope and this results in an overall smaller structure. The effect of the nozzle moving speed on the growth of the electrospun branches is illustrated in the ESI (Fig. S13†).

**Fig. 17:**
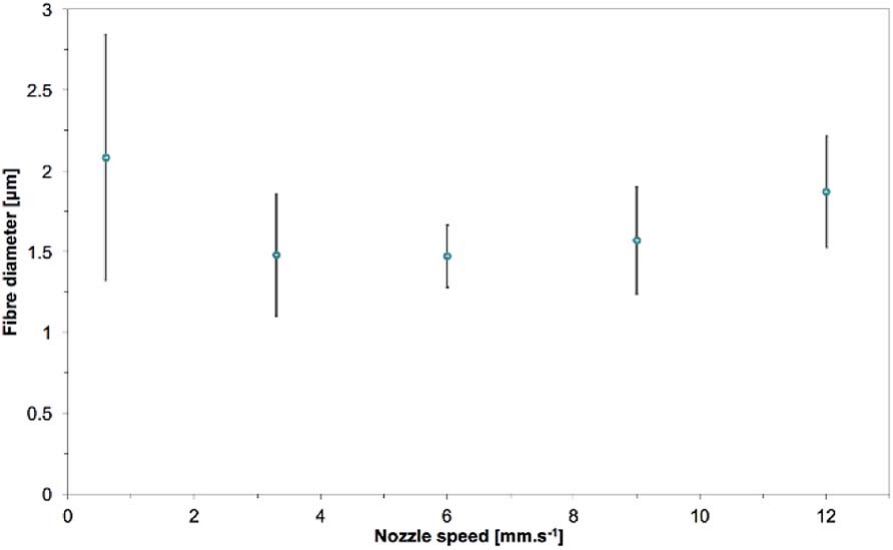
Evolution of the mean fiber diameters as the nozzle speed is increased from 0.6 to 12.0 mm s^−1^. 15.0 wt% PS in 1 : 1 DMF/THF was electrospun at a voltage of +20 kV, a working distance of 5 cm and a flow rate of 5.0 mL h^−1^ for 10 minutes. Low nozzle speed is detrimental to the drying and thus stretching of the fibers, resulting in bigger fibers.

The mean fiber diameters were 2.08 ± 0.76, 1.48 ± 0.38, 1.47 ± 0.19, 1.57 ± 0.33, 1.87 ± 0.34 μm for nozzle speeds of 0.6, 3.3, 6.0, 9.0, 12.0 mm s^−1^ respectively (see Fig. 17). The largest fiber diameters were obtained for the slowest nozzle speed and even fused fibers were observed. A slow nozzle speed would increase the amount of polymer solution in a single position, thus decrease the drying speed and yield bigger fiber diameters. This effect is similar to increasing the flow rate. All the other samples electrospun at different increasing nozzle moving speeds have similar fibers diameter. In that range of speed, a moving nozzle has no direct influence on the jet elongation and drying except from spreading the polymer jet over a wider area on the collector. It is interesting to note that using a moving nozzle enables electrospinning of individual fibers at high flow rate.

### Nozzle pattern and shape of the electrospun 3D structure

**Fig. 18:**
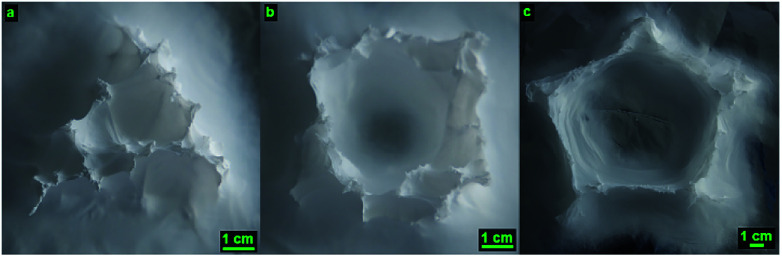
Different 3D structures electrospun with the optimal parameters: 15.0 wt% PS in 1 : 1 DMF/THF electrospun at a voltage of +15–20 kV, a working distance of 5 cm, a flow rate of 5.0 mL h^−1^ and a nozzle speed of 12 mm s^−1^ for 10 minutes. (a) Triangle. (b) Square. (c) Five-pointed star polygon.

Different nozzle patterns have been tested to prove the versatility of this technique to build different 3D shaped structures. An equilateral triangle with a side length of 5 cm, a square with a side length of 5 cm as well as a five-pointed star polygon with a diameter of 7.5 cm have been electrospun using the optimal parameters as investigated previously (concentration 15 wt% PS, applied voltage 15–20 kV, working distance 5 cm, flow rate 5 mL h^−1^, nozzle speed 12 mm s^−1^). Fig. 18 depicts the resulting shapes after electrospinning for 10 minutes. The square shape resembled the CAD file the most accurately, having right angles and a hollow inside. Its shape, along with the one of the cylinder, was the closest to the designed nozzle pattern. The triangle shape was completely filled, this is due to the close proximity between the 3 segments of the triangle and the relatively large deposition area of the electrospun structure. The electrospun fibers from the previous segment can act as a preferential deposition area and attract some fibers toward it. In effect, there is a bridging effect between 2 segments, which fills the triangle. A similar effect is observed in the five-pointed star, where all 5 corners are made of small filled triangles, but the shape of the star is still followed. The original gcode file of these 3 shapes can be seen in the ESI S14.† Overall, this technique has proved successful in its flexibility to electrospin different 3D structures in a short time.

### Possible extension to other polymers

A few other polymers were investigated as well for 3D build-up. Li and Long have successfully electrospun a non-organized 3D structure of polyvinylpyrrolidone (PVP) by using a self-assembly mechanism,^41^ which is closely related to the 3D build-up investigated in this paper. In a similar fashion, M. Yousefzadeh *et al.* managed to electrospin a “fluffy web” of polyacrylonitrile (PAN).^70^ In both research, increasing the conductivity of the solution with additives was key to enable the self-assembly mechanism. However, preliminary research of 3D electrospinning of PVP and PAN solutions have failed to yield any buildup. Reasons for the non-reproducibility could be attributed to the different nature of the additives used, which would yield different properties of the final polymer solution.

## Conclusions

This study explored the use of a 3D electrospinner in the fabrication of 3D fibrous architecture. Rapid electrospinning of designed 3D structures with controllable shape has been successfully achieved without the aid of any auxiliary template. The electrospun 3D structures have typical height of approximately 3–4 cm from a 10 minutes electrospinning process and are self-standing even after 6 months of storage at ambient condition. The build-up of the 3D fibrous polystyrene structure is associated with the rapid solidification of the fibers, the charge induction and polarization of the fibers, and their interaction with the electrospinning environment, including the charged nozzle. The behavior of the electrospun fibrous 3D structure in the vicinity of a charged rod, during and after electrospinning, has been investigated and goes in line with this theory. Proper tuning of the process parameters, including solution concentration, applied voltage, working distance, flow rate and nozzle moving speed, is critical to achieve a 3D build-up instead of the traditional 2D deposition in electrospinning. The parameters must be adjusted to control the size of the deposition area, to control the speed of the vertical fibers growth and to provide a decent amount of polymer, high enough to have enough repulsion between fibers and low enough for proper drying of the fibers. The PS mean fiber diameters of the 3D structures are between 550 nm to 2.89 μm, the evolution of the fibers morphology and diameter with the process parameters is similar in behavior with the fibers obtained with a traditional 2D electrospinner. 3D electrospinning technology opens new horizons in nano- and micro-fabrication, notably for the fast and facile fabrication of controllable scaffold for bio-engineering applications.

## Conflicts of interest

There are no conflicts to declare.

## Supplementary Material

RA-008-C7RA13278F-s001

RA-008-C7RA13278F-s002

RA-008-C7RA13278F-s003
