# Supplementary material for: Controlled three-dimensional polystyrene micro- and nano-structures fabricated by three-dimensional electrospinning
Source: RSC Adv. 2018 Apr 24;8(28):15501–12. doi: 10.1039/c7ra13278f (PMC9080079; doi:10.1039/c7ra13278f)
Supplement: RA-008-C7RA13278F-s003 [file RA-008-C7RA13278F-s003.docx]

**Fig. S1** Side view of the growth process of the electrospun PS fibers 3D structure minute by minute. The electrospinning was performed at a voltage of +12 kV, a working distance of 5 cm, a flow rate of 5.0 mL.h^-1^ and at a nozzle moving speed of 12.0 mm.s^-1^ for 10 minutes. The 3D build-up starts after an initial flat layer of fibers have been deposited onto the collector as seen in a. The build-up follows the circular pattern of the nozzle, forming a hollow cylinder.

**Fig. S2** Top view of the growth process of the electrospun PS fibers 3D structure minute by minute. The electrospinning was performed at a voltage of +12 kV, a working distance of 5 cm, a flow rate of 5.0 mL.h^-1^ and at a nozzle moving speed of 12.0 mm.s^-1^ for 10 minutes. The wall thickness of the electrospun cylinder increases over time.

**Fig. S3** Evolution of the 3D structures shape and the fibers shape and morphology as the weight concentration of PS is increased from 5.0 wt% to 15.0 wt%. The solutions were electrospun at a voltage of +20 kV, a working distance of 5 cm, a flow rate of 5.0 mL.h^-1^ and at a nozzle speed of 12.0 mm.s^-1^ for 10 minutes. No 3D build-up were observed at low polymer concentration. The mean fiber diameter decreased with decreasing concentration. Presence of beads was observed at low concentration.

**Fig. S4** Comparison of the electric field intensity in the y-z plane. The maximum electric field strength generated from the needle at an applied voltage of (a) 7kV, (b) 15kV and (c) 20kV at a working distance of 5 cm are 9.01 x 106, 1.93 x 107 and 2.57 x 107 V.m^-1^. This simulation shows how the electrostatic field strength increases with increasing applied voltage at a constant working distance. The dark red region shows the region with an electric field strength greater than 30000 V.m^-1^. The electric field magnitude across the collector also increases with applied voltage.

**Fig. S5** Evolution of the 3D structures shape and the fibers shape and morphology as the voltage is increased from +7 kV to +11 kV. 15.0 wt% PS in 1:1 DMF/THF was electrospun at a working distance of 5 cm, a flow rate of 5.0 mL.h^-1^, at a nozzle moving speed of 12.0 mm.s^-1^ for 10 minutes. The sample processed at +10 kV had the closest representation to the designed cylinder, having thin walls and the least amount of fibers coverage inside the cylinder. Other than that, the applied voltage had little influence on the cylinder shape. The mean fiber diameters had no linear correlation with the applied voltage.

**Fig. S6** Evolution of the 3D structures shape and the fibers shape and morphology as the voltage is increased from +12 kV to +16 kV. 15.0 wt% PS in 1:1 DMF/THF was electrospun at a working distance of 5 cm, a flow rate of 5.0 mL.h^-1^, at a nozzle moving speed of 12.0 mm.s^-1^ for 10 minutes.

**Fig. S7** Evolution of the 3D structures shape and the fibers shape and morphology as the voltage is increased from +17 kV to +20 kV. 15.0 wt% PS in 1:1 DMF/THF was electrospun at a working distance of 5 cm, a flow rate of 5.0 mL.h^-1^, at a nozzle moving speed of 12.0 mm.s^-1^ for 10 minutes.

**Fig. S8** Evolution of the 3D structures shape and the fibers shape and morphology at low working distance 1 and 2 cm. 15.0 wt% PS in 1:1 DMF/THF was electrospun at a voltage of +20 kV, a flow rate of 5.0 mL.h^-1^, at a nozzle moving speed of 12.0 mm.s^-1^ for 10 minutes. At low working distances and ambient conditions, flight time of the electrospun jet was insufficient and fibers were fused together.

**Fig. S9** Evolution of the 3D structures shape and the fibers shape and morphology as the working distance is increased from 3 cm to 7 cm. 15.0 wt% PS in 1:1 DMF/THF was electrospun at a voltage of +20 kV, a flow rate of 5.0 mL.h^-1^, at a nozzle moving speed of 12.0 mm.s^-1^ for 10 minutes. At intermediate working distance, the cylinder shape is observed and the 3D structures are made of individual fibers.

**Fig. S10** Evolution of the 3D structures shape and the fibers shape and morphology as the working distance is increased from 8 cm to 10 cm. 15.0 wt% PS in 1:1 DMF/THF was electrospun at a voltage of +20 kV, a flow rate of 5.0 mL.h^-1^, at a nozzle moving speed of 12.0 mm.s^-1^ for 10 minutes. In this particular setup, high working distance is detrimental to the overall shape of the electrospun cylinder.

**Fig. S11** Evolution of the 3D structures shape and the fibers shape and morphology as the flow rate is increased from 1.0 mL.h^-1^ to 4.0 mL.h^-1^. 15.0 wt% PS in 1:1 DMF/THF was electrospun at a voltage of +20 kV, a working distance of 5 cm, at a nozzle moving speed of 12.0 mm.s^-1^ for 10 minutes. A minimum flow rate is necessary to allow 3D build-up to happen.

**Fig. S12** Evolution of the 3D structures shape and the fibers shape and morphology as the flow rate is increased from 1.0 mL.h^-1^ to 10.0 mL.h^-1^. 15.0 wt% PS in 1:1 DMF/THF was electrospun at a voltage of +20 kV, a working distance of 5 cm, at a nozzle moving speed of 12.0 mm.s^-1^ for 10 minutes. High flow rate hinders the controlled build-up of the 3D structure, notably in the morphology of the fibers.

**Fig. S13** Simplified explanation on the effect of the nozzle speed on the 3D structure formation. a) With an immobile nozzle (v_a_ = 0 mm.s^-1^), the fibers grow up as a single branch until they touch the nozzle at which point dripping occurs. b) With an intermediate nozzle moving speed (v_b_ ≠ 0 mm.s^-1^), the fibers branches can grow high before settling onto the collector. c) At a high moving speed (v_c_ >> v_b_), the branches are forced along a smaller slope which lead to a smaller structure.
